# Supplementary material for: Long-Term Persistence of Mitochondrial DNA Instability in HIV-Exposed Uninfected Children during and after Exposure to Antiretroviral Drugs and HIV
Source: Biomedicines. 2022 Jul 25;10(8):1786. doi: 10.3390/biomedicines10081786 (PMC9331317; doi:10.3390/biomedicines10081786)
Supplement: Supplementary file 1 [file biomedicines-10-01786-s001.zip › biomedicines-1785715-supplementary.pdf]

Supplementary tables

Table S1. Comparison of randomly selected children from Burkina Faso and Zambia that were not included in the analysis (n=87) and those that were (n=24) .

Characteristics		Included (n=24)	Not included (n=87)	p-value
**Socio-demographics**				
Site; *n (%)*				0.930
	Burkina Faso	13 (54.2)	48 (55.2)	
	Zambia	11 (45.8)	39 (44.8)	
Sex; *n (%)*				
	Boy	12 (50.0)	47 (54.0)	0.727
**Anthropometrics**; *mean ± SD*				
Weight (kg)		3.1 ± 0.4	3.1 ± 0.4	0.793
Height (cm)		49.0 ± 2.2	49.1 ± 1.7	0.763
WAZ		-0.7 ± 0.9	-0.7 ±0.9	0.828
HAZ		-0.9 ± 1.1	-0.9 ± 0.9	0.812
WHZ		-0.4 ± 1.5	-0.3 ± 1.0	0.789
Gestational age (week); *median [IQR]*		38 [37-39.5]	38 [36-39]	0.454
Preterm birth (week); *n (%)*				
	No prematurity ≥ 37	21 (87.5)	61 (70.1)	0.116*
**Hematology**; *n (%)*				
Hemoglobin concentration (g/dL); n (%)				1.000*
	Normal	22 (91.7)	80 (92.0)	
	Anemia	2 (8.3)	7 (8.0)	
Platelet count (10^3/mm^3); n (%)				1.000*
	Normal >125	24 (100.0)	86 (99.0)	
Leucocyte count (10^3/mm^3); n (%)				N.A.
	Normal >2.5	24 (100.0)	87 (100.0)	
Neutrophil count (10^3/mm^3); n (%)				0.578*
	Normal	23 (100.0)	83 (95.4)	
	Neutropenia [¥]	0 (0.0)	4 (4.6)	
**Biochemistry**; *n (%)*				
ALT concentration (U/L)				0.337*
	Normal < 1.25xULN	24 (100.0)	81 (93.1)	
	Abnormal ≥ 1.25xULN	0 (0.0)	6 (6.9)	
**Mother's characteristics**				
Age (year); *mean ± SD*		28.2 ± 5.6	28.5 ± 5.2	0.811
Parity; *median [IQR]*		3 [2-4]	3 [1.5-3]	0.387
Mother/caregiver ever attended school; *n (%)*				
	Yes	18 (75.0)	61 (70.1)	0.640
BMI; *mean ± SD*		23.3 ± 2.8	23.9 ± 4.0	0.479
HIV viral load; *n (%)*				0.566
	≤1000 copies/mL	14 (58.3)	45 (51.7)	
	>1000 copies/mL	10 (41.7)	42 (48.3)	
ARV regimen; *n (%)*				1.000*
	containing AZT	24 (100.0)	85 (97.7)	
Duration of ARV prophylaxis (week); *median [IQR]*		8 [4-9]	8 [5-11]]	0.088
Smoking during pregnancy; *n (%)†*				N.A.
	No	24 (100.0)	56 (100.0)	

Alcohol consumption during pregnancy; *n (%)†*			0.013
Yes	12 (54.6)	14 (25.0)	

*p-value obtained from Fischer’s exact test; ‡Variables from PROMISE M&S (Y6), 21 missing values. Abbreviations: SD, standard deviation; kg, kilogram; cm, centimetres; WAZ, weight-for-age Z-score; HAZ, height-for-age Z-score; IQR, interquartile range; ALT, alanine transaminase; BMI, body mass index; HIV, human immunodeficiency virus; mL, millilitres; ARV, antiretroviral.

Table S2. List of mtDNA alterations.

Participant Id	Time point	Alteration event	5' breakpoint	3' breakpoint	heteroplasmy (%)	Flanking repeat at break
#1	D7	Del	6516	4201	0,96	6517-GCAT-6520 <> 4201-GCAT-4204
#1	D7	Del	6843	4057	1,88	6844-TCCCC-6848 <> 4057-TCCCC-4061
#1	D7	Del	7034	14400	1,78	7035-CACT-7038 <> 14400-CACT-14403
#1	D7	Del	15024	4830	0,13	none
#1	W50	Del	7318	11257	0,12	none
#1	W50	Del	9387	14783	5,51	9385-TG-9386 <> 14782-TG-14783
#1	Y6	Del	6333	15830	0,07	6334-A-6334 <> 15830-A-15830
#1	Y6	Del	8000	14573	0,06	8001-ACAATC-8006 <> 14573-ACAATC-14578
#1	Y6	Del	8869	14844	0,55	8867-TA-8868 <> 14843-TA-14844
#1	D7	Dupl	4254	7122	0,13	4255-CAAACCTA-4262 <> 7122-CAAACCTA-7129
#1	D7	Dupl	14857	6891	0,09	none
#2	D7	Del	7033	14400	0,10	7034-C-7034 <> 14400-C-14400
#2	W50	Del	6485	3553	2,37	6486-CTTCT-6490 <> 3553-CTTCT-3557
#2	W50	Del	7034	14400	0,15	7035-CACT-7038 <> 14400-CACT-14403
#2	W50	Del	7318	13373	4,53	7319-T-7319 <> 13373-T-13373
#2	W50	Del	7526	14710	0,50	7527-GAAAAACCAT-7536 <> 14710-GAAAAACCAT-14719
#2	Y6	Del	1572	1605	0,17	1573-AGTGTA-1578 <> 1605-AGTGTA-1610
#4	W50	Del	7707	15025	3,12	7708-CCT-7710 <> 15025-CCT-15027
#4	D7	Dupl	15045	7017	4,61	none
#4	W50	Dupl	15045	7017	0,14	none
#5	W50	Del	7569	4049	0,16	7570-A-7570 <> 4049-A-4049
#5	W50	Del	15276	5313	0,20	15277-AT-15278 <> 5313-AT-5314
#5	Y6	Del	6430	3702	0,70	6428-AT-6429 <> 3701-AT-3702
#6	D7	Del	6923	3967	0,88	6924-GCCCTA-6929 <> 3967-GCCCTA-3972
#6	D7	Del	7707	15025	1,88	7708-CCT-7710 <> 15025-CCT-15027
#6	W50	Del	7034	14400	0,11	7035-CACT-7038 <> 14400-CACT-14403
#6	W50	Dupl	14857	6891	0,06	none

#6	Y6	Del	8279	14641	0,21	8280-ACCC-8283 <> 14641-ACCC-14644
#7	D7	Dupl	14857	6891	0,12	none
#7	D7	Dupl	15117	5942	11,06	15118-AG-15119 <> 5942-AG-5943
#7	D7	Dupl	15686	6570	5,24	15687-GC-15688 <> 6570-GC-6571
#7	W50	Dupl	15321	6476	0,07	15322-AGCA-15325 <> 6476-AGCA-6479
#7	D7	Del	5956	4225	0,59	5957-ATACC-5961 <> 4225-ATACC-4229
#7	D7	Del	7034	14400	0,32	7035-CACT-7038 <> 14400-CACT-14403
#7	D7	Del	9418	14699	39,01	none
#7	W50	Del	7285	14225	0,08	none
#7	W50	Del	8257	14421	0,28	none
#7	Y6	Del	7515	14012	0,39	7516-AAAAG-7520 <> 14012-AAAAG-14016
#8	D7	Del	7034	14400	0,26	7035-CACT-7038 <> 14400-CACT-14403
#8	D7	Del	7091	3716	0,15	none
#8	W50	Del	7034	14400	0,06	7035-CACT-7038 <> 14400-CACT-14403
#8	W50	Del	7114	13992	0,06	7115-CCTAGACC-7122 <> 13992-CCTAGACC-13999
#8	D7	Dupl	14857	6888	0,05	14858-GG-14859 <> 6888-GG-6889
#9	D7	Dupl	4992	14757	0,26	4993-TACGCAAAA-5001 <> 14757-TACGCAAAA-14765
#9	D7	Del	7034	14400	0,16	7035-CACT-7038 <> 14400-CACT-14403
#9	Y6	Del	7122	13096	0,20	none
#9	Y6	Del	9329	14827	0,10	9330-CTCC-9333 <> 14827-CTCC-14830
#9	Y6	Dupl	1621	6080	0,05	1617-ACAA-1620 <> 6077-ACAA-6080
#9	Y6	Dupl	14962	6755	0,47	14963-GT-14964 <> 6755-GT-6756
#10	D7	Del	7919	14732	1,93	7920-ACTAC-7924 <> 14732-ACTAC-14736
#10	W50	Del	7042	14953	0,32	7043-C-7043 <> 14953-C-14953
#10	W50	Del	7726	5135	0,10	7724-CA-7725 <> 5134-CA-5135
#10	W50	Del	7749	14400	0,10	7750-C-7750 <> 14400-C-14400
#10	W50	Del	15043	5135	1,27	15044-C-15044 <> 5135-C-5135
#10	D7	Dupl	15052	7017	0,26	15053-TA-15054 <> 7017-TA-7018
#10	W50	Dupl	5198	7017	0,44	5197-A-5197 <> 7017-A-7017
#10	W50	Dupl	15048	7017	6,99	none
#10	W50	Dupl	15061	14050	0,53	15062-TC-15063 <> 14050-TC-14051
#10	W50	Dupl	15079	9312	0,32	none
#10	Y6	Dupl	16095	7130	0,13	none
#11	D7	Dupl	15827	6950	0,06	15828-C-15828 <> 6950-C-6950
#11	W50	Dupl	14857	6891	0,08	none
#11	W50	Del	7034	14400	1,10	7035-CACT-7038 <> 14400-CACT-14403

#11	Y6	Del	7707	15025	0,63	7708-CCT-7710 <> 15025-CCT-15027
#11	Y6	Del	7838	14933	2,53	7837-T-7837 <> 14933-T-14933
#12	W50	Del	7032	14400	0,16	7030-CT-7031 <> 14399-CT-14400
#12	Y6	Del	7144	14568	0,07	7145-C-7145 <> 14568-C-14568
#12	Y6	Del	7770	14884	0,07	none
#12	W50	Dupl	16028	7056	0,12	none
#13	W50	Del	10372	3537	0,65	none
#13	Y6	Del	6324	3692	0,13	none
#13	Y6	Del	6349	3273	0,46	6348-C-6348 <> 3273-C-3273
#13	Y6	Del	7094	13981	2,36	7095-CCCCTA-7100 <> 13981-CCCCTA-13986
#14	W50	Dupl	14991	6669	0,25	14989-CT-14990 <> 6668-CT-6669
#15	D7	Del	7034	14400	0,23	7035-CACT-7038 <> 14400-CACT-14403
#15	D7	Del	7707	15025	0,86	7708-CCT-7710 <> 15025-CCT-15027
#15	W50	Del	7034	14400	0,07	7035-CACT-7038 <> 14400-CACT-14403
#15	W50	Del	7707	15025	0,36	7708-CCT-7710 <> 15025-CCT-15027
#15	W50	Dupl	772	14969	1,15	773-T-773 <> 14969-T-14969
#15	W50	Dupl	15291	6104	0,10	15292-CTTCAT-15297 <> 6104-CTTCAT-6109
#15	W50	Dupl	15503	7007	0,38	15501-CC-15502 <> 7006-CC-7007
#15	Y6	Dupl	15684	6657	0,30	none
#16	W50	Dupl	15043	7017	1,68	none
#16	W50	Dupl	15348	7144	0,29	none
#16	D7	Dupl	678	14812	3,13	679-C-679 <> 14812-C-14812
#16	W50	Del	7747	14400	0,63	none
#16	W50	Del	15171	322	0,26	15172-GGCCACAG-15179 <> 322-GGCCACAG-329
#16	Y6	Dupl	15017	6067	0,18	none
#16	Y6	Dupl	15143	6393	0,14	15144-T-15144 <> 6393-T-6393
#17	W50	Del	6100	3216	0,28	6101-C-6101 <> 3216-C-3216
#17	W50	Del	6302	3939	0,08	6301-A-6301 <> 3939-A-3939
#17	W50	Del	7707	15025	0,13	7708-CCT-7710 <> 15025-CCT-15027
#17	Y6	Dupl	15148	7034	1,55	15147-G-15147 <> 7034-G-7034
#18	Y6	Del	7356	14610	0,04	7357-TAGAAGAA-7364 <> 14610-TAGAAGAA-14617
#18	Y6	Del	8940	14642	0,02	8941-CCCA-8944 <> 14642-CCCA-14645
#18	D7	Dupl	14857	6888	0,02	14858-GG-14859 <> 6888-GG-6889
#19	D7	Del	7034	14400	0,27	7035-CACT-7038 <> 14400-CACT-14403
#19	W50	Del	7034	14400	0,06	7035-CACT-7038 <> 14400-CACT-14403
#19	W50	Del	7321	14910	1,60	7322-A-7322 <> 14910-A-14910
#19	Y6	Dupl	16491	6016	0,10	16490-T-16490 <> 6016-T-6016

#20	W50	Del	7034	14400	0,43	7035-CACT-7038 <> 14400-CACT-14403
#20	Y6	Del	7707	15025	0,19	7708-CCT-7710 <> 15025-CCT-15027
#20	D7	Dupl	14928	7139	0,10	14929-C-14929 <> 7139-C-7139
#21	W50	Dupl	14857	6891	0,04	none
#21	Y6	Dupl	15303	6446	0,25	none
#21	W50	Del	7034	14400	0,22	7035-CACT-7038 <> 14400-CACT-14403
#21	Y6	Del	7543	12688	0,23	none
#21	Y6	Del	7952	14805	0,12	7951-C-7951 <> 14805-C-14805
#22	D7	Dupl	14935	7047	14,79	14936-TCA-14938 <> 7047-TCA-7049
#22	D7	Dupl	14943	7056	0,06	none
#22	Y6	Dupl	716	5916	0,10	none
#22	D7	Del	293	344	0,26	294-TC-295 <> 344-TC-345
#22	D7	Del	7237	12133	84,59	none
#22	Y6	Del	7034	14400	0,18	7035-CACT-7038 <> 14400-CACT-14403
#23	W50	Del	5810	3501	57,66	5811-A-5811 <> 3501-A-3501
#23	Y6	Del	8269	12792	0,24	8270-C-8270 <> 12792-C-12792
#23	Y6	Del	7034	14400	0,14	7035-CACT-7038 <> 14400-CACT-14403
#23	Y6	Del	7040	13770	0,17	none
#24	D7	Del	7034	14400	0,36	7035-CACT-7038 <> 14400-CACT-14403

Table S3. Association between health outcomes at Y6 and having a heteroplasmy rate >1% at D7 or W50.

**Health outcomes at Y6**	**Cumulative heteroplasmy**		p-value
	**rate >1% at D7 or W50** n=12	**rate ≤1% at D7 or W50** n=12	
**Growth** *mean ± SD*			
Head circumference (cm)	50.78 ± 1.72	50.91 ± 1.73†	0.849
WAZ	-1.08 ± 0.67	-0.43 ± 1.19	0.111
HAZ	-0.77 ± 0.91	-0.36 ± 1.15	0.353
BMIZ	-0.92 ± 0.79	-0.29 ± 1.02	0.104
**Clinical evaluation**			
Systolic blood pressure Z-score; *mean ± SD*	0.46 ± 0.94	0.38 ± 1.15	0.840
Clinical abnormalities; *n (%)*	1 (8.33)	3 (25.0)	0.590
Hospitalised since PROMISE PEP; *n (%)*	3 (25.0)	2 (16.67)	1.000
Abnormal LDH concentration; *n (%)*	9 (75.0)	8 (66.67)	1.000
Abnormal ALT concentration; *n (%)*	0 (0.0)	0 (0.0)	N.A.
**Developmental outcomes**			
At least one disability detected from the 10questionPlus; *n (%)*	4 (33.33)	3 (25.0)	1.000
SDQ-25; *mean ± SD*	8.75 ± 3.96	6.67 ± 4.91	0.265
TOVA; *mean ± SD*	2.09 ± 0.58	2.38 ± 0.77†	0.321
MABC-2; *mean ± SD*	74.64 ± 8.92†	76.55 ± 10.54†	0.652

KABC-II; *mean ± SD*	52.91 ± 16.81†	61.91 ± 8.61†	0.130

†one missing value. Abbreviations: SD, standard deviation; WAZ, weight-for-age Z-score; HAZ, height-for-age Z-score; BMIZ, paediatric body mass index Z-score; LDH, Lactate dehydrogenase; ALT, alanine transaminase; SDQ-25, Strengths and Difficulties Questionnaire; TOVA, Test of Variable of Attention; MABC-2, Movement Assessment Battery for Children second edition; KABC-II, Kaufman Assessment Battery for Children second edition.

Supplementary Table S4: Association between mother's viral load at birth (<1000 versus ≥1000 cp/ml) and poor health outcomes at Y6, stratified by heteroplasmy rate (>1% or ≤1%) at D7

**Health outcomes at Y6**	**Linear regression coefficient (β) or Relative Risk (RR) and 95%CI for mother's viral load at birth, by cumulative heteroplasmy rate**		p-value for interaction
	**> 1% at D7** n=7	**≤ 1% at D7** n=17	
**Growth**			
Head circumference (cm)	0.11 [-3.55 – 3.78]	0.09 [-1.86 – 2.04]†	0.990
WAZ	0.56 [-0.74 – 1.86]	-0.50 [-1.50 – 0.51]	0.250
HAZ	1.28 [-0.07 -2.62]	0.06 [-0.99 – 1.11]	0.206
BMIZ	-0.55 [-2.48 – 1.37]	-0.85 [-1.75 – 0.05]	0.713
**Clinical evaluation**			
Systolic blood pressure Z-score	1.18 [-0.34 – 2.70]	0.10 [-0.96 – 1.17]	0.266
LDH concentration	6.17 [-354.68 – 367.01]	-172.23 [-598.51 – 253.51]	0.640
ALT concentration	-8.17 [-31.01 – 14.68]	1.36 [-6.65 – 9.37]	0.194

†one missing value. Abbreviations: SD, standard deviation; WAZ, weight-for-age Z-score; HAZ, height-for-age Z-score; BMIZ, paediatric body mass index Z-score; LDH, Lactate dehydrogenase; ALT, alanine transaminase.

Table S5. Association between infant WHZ at D7 and poor health outcomes at Y6, stratified by heteroplasmy rate (>1% or ≤1%) at D7.

**Health outcomes at Y6**	**Linear regression coefficient (β) or Relative Risk (RR) and 95%CI for mother's age at birth, by cumulative heteroplasmy rate**		p-value for interaction
	**> 1% at D7** n=7	**≤ 1% at D7** n=17	
**Growth**			
Head circumference (cm)	0.60 [-0.58 – 1.77]	0.32 [-0.26 – 0.90]†	0.647
WAZ	0.08 [-0.38 – 0.55]	-0.11 [-0.46 – 0.24]	0.596
HAZ	-0.33 [-1.07 – 0.40]	-0.14 [-0.50 – 0.23]	0.613
BMIZ	0.49 [-0.27 – 1.24]	-0.02 [-0.35 – 0.31]	0.153
**Clinical evaluation**			
Systolic blood pressure Z-score	-0.44 [-1.19 – 0.31]	-0.10 [-0.47 – 0.27]	0.388
LDH concentration	29.33 [-151.13 – 209.78]	157.78 [25.38 – 290.18]	0.361
ALT concentration	-1.39 [-10.34 – 7.56]	-1.14 [-3.56 – 1.28]	0.922

†one missing value. Abbreviations: SD, standard deviation; WAZ, weight-for-age Z-score; HAZ, height-for-age Z-score; BMIZ, paediatric body mass index Z-score; LDH, Lactate dehydrogenase; ALT, alanine transaminase.

Supplementary Table S1:

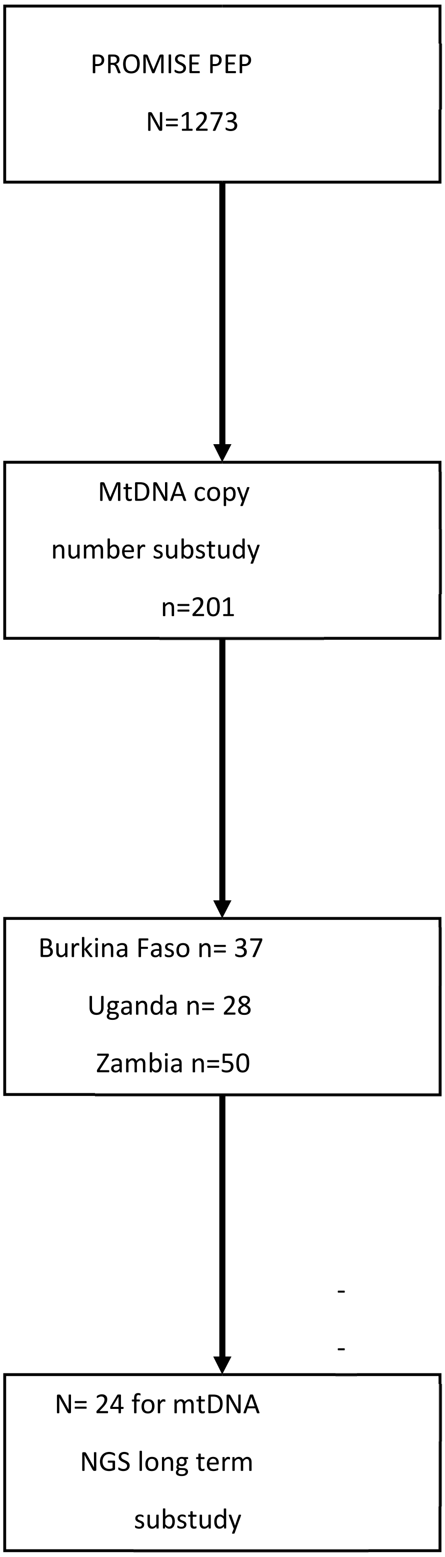

Random selection with the following criteria - 50 children per country

- Ratio M/F = 1/1
- Ratio 3TC/LPVr = 1/1

- recaptured in PROMISE M&S

N.B.: South Africa had not biobank

Random selection with the following criteria - 12 children per country Ratio M/F = 1/1

-
- Ratio 3TC/LPVr = 1/1

N.B.: To keep numbers high enough, Uganda was not part of the selection

Figure S1. Sample selection flow chart. Out of the children enrolled in the RPOMISE-PEP trial in 20092012, 201 were randomly selected for a first substudy on mitochondrial parameters among HEU (Monnin et al., 2019). Out of these 201, 115 were recaptured during the PROMISE-M&S trial conducted in 2017-2018.
